# First experience with ^224^Radium-labeled microparticles (Radspherin®) after CRS-HIPEC for peritoneal metastasis in colorectal cancer (a phase 1 study)

**DOI:** 10.3389/fmed.2023.1070362

**Published:** 2023-03-01

**Authors:** Stein Gunnar Larsen, Wilhelm Graf, Anthony Burton Mariathasan, Olaf Sørensen, Milan Spasojevic, Mariusz Adam Goscinski, Silje Selboe, Nadja Lundstrøm, Anne Holtermann, Mona-Elisabeth Revheim, Øyvind Sverre Bruland

**Affiliations:** ^1^Department of Gastroenterological Surgery, Section for Surgical Oncology, Norwegian Radium Hospital, Oslo University Hospital, Oslo, Norway; ^2^Department of Surgical Sciences, Uppsala University, Uppsala, Sweden; ^3^Uppsala Academic Hospital, Uppsala, Sweden; ^4^Division of Radiology and Nuclear Medicine, Oslo University Hospital, Oslo, Norway; ^5^Department of Nuclear Medicine, Uppsala, Sweden; ^6^Institute for Clinical Medicine, Faculty of Medicine, University of Oslo, Oslo, Norway; ^7^Department of Oncology, Oslo University Hospital, Oslo, Norway; ^8^Oncoinvent AS, Oslo, Norway

**Keywords:** metastatic colorectal cancer, peritoneal metastasis, cytoreductive surgery, hyperthermic intraperitoneal chemotherapy, ^224^Ra, alpha emitter, targeted alpha particle therapy

## Abstract

**Background:**

Peritoneal metastasis (PM) from colorectal cancer carries a dismal prognosis despite extensive cytoreductive surgery and hyperthermic intraperitoneal chemotherapy (CRS-HIPEC). With a median time to recurrence of 11–12 months, there is a need for novel therapies. Radspherin® consists of the α-emitting radionuclide radium-224 (^224^Ra), which has a half-life of 3.6 days and is adsorbed to a suspension of biodegradable calcium carbonate microparticles that are designed to give short-range radiation to the serosal peritoneal surface linings, killing free-floating and/or tumor cell clusters that remain after CRS-HIPEC.

**Methods:**

A first-in-human phase 1 study (EudraCT 2018–002803-33) was conducted at two specialized CRS-HIPEC centers. Radspherin® was administered intraperitoneally 2 days after CRS-HIPEC. Dose escalation at increasing activity dose levels of 1-2-4-7-MBq, a split-dose repeated injection, and expansion cohorts were used to evaluate the safety and tolerability of Radspherin®. The aim was to explore the recommended dose and biodistribution using gamma-camera imaging. The results from the planned safety interim analysis after the completion of the dose-limiting toxicity (DLT) period of 30 days are presented.

**Results:**

Twenty-three patients were enrolled: 14 in the dose escalation cohort, three in the repeated cohort, and six in the expansion cohort. Of the 23 enrolled patients, seven were men and 16 were women with a median age of 64 years (28–78). Twelve patients had synchronous PM stage IV and 11 patients had metachronous PM [primary stage II; (6) and stage III; (5)], with a disease-free interval of 15 months (3–30). The peritoneal cancer index was median 7 (3–19), operation time was 395 min (194–515), and hospital stay was 12 days (7–37). A total of 68 grade 2 adverse events were reported for 17 patients during the first 30 days; most were considered related to CRS and/or HIPEC. Only six of the TEAEs were evaluated as related to Radspherin®. One TEAE, anastomotic leakage, was reported as grade 3. Accordion ≥3 grade events occurred in a total of four of the 23 patients: reoperation due to anastomotic leaks (two) and drained abscesses (two). No DLT was documented at the 7 MBq dose level that was then defined as the recommended dose. The biodistribution of Radspherin® showed a relatively even peritoneal distribution.

**Conclusion:**

All dose levels of Radspherin® were well tolerated, and DLT was not reached. No deaths occurred, and no serious adverse events were considered related to Radspherin®.

**Clinical Trial Registration:** Clinicaltrials.gov, NCT 03732781.

## Introduction

Peritoneal metastasis from colorectal cancer (CRC) carries a worse prognosis than hepatic and lung metastases (1). Most patients with metastatic CRC (mCRC) cannot be cured, illustrated by a 5-year survival of 10–20% in study patients (2, 3), and with an even more grim prognosis in population-based registries reporting a median survival of 5–12 months and 5-year survival of 5–10% (4, 5). The incidence of peritoneal metastasis (PM) is approximately 4–10% at the time of diagnosis and 4–12% in patients with recurrence after primary curative resection (6–8).

In cases with limited peritoneal tumor load, improved and even long-term survival can be achieved by combining complete cytoreductive surgery (CRS) and hyperthermic intraperitoneal chemotherapy (HIPEC) as shown in a randomized controlled trial (9), case–control studies (10–12), meta-analysis (13), and several cohort studies (14, 15). Systemic chemotherapy alone has a limited effect on localized PM-CRC with a median survival of 13–16 months (1, 16). CRS-HIPEC aims to remove all macroscopic tumors and achieve high intraperitoneal concentrations of hyperthermic cytotoxic drugs (17).

The outcome of CRS-HIPEC is, however, highly variable, and most patients will experience disease recurrence with a 5-year overall survival (OS) reported in about 40% of CRS-HIPEC cases (13, 15). However, the 5-year disease-free survival (DFS) is only 18% with a median time to relapse between 11 and 12 months. At the moment of recurrence, two-thirds of patients suffer either from peritoneal relapse or peritoneal relapse and distant metastases together (18).

If PM recurrence after CRS-HIPEC occurs, the prognosis is dismal. Hence, there is a definite unmet medical need for novel treatments against abdominal cancer dissemination and novel therapeutic strategies that may help preserve the surgical complete response after CRS-HIPEC.

Intraperitoneal (IP) therapy with α-emitters may be beneficial for patients with PM-CRC since hallmarks of the disease include dissemination within the abdominal cavity and residual micrometastases in a substantial number of patients. Preclinical studies have tested α-emitting radioimmunoconjugates as IP treatment of ovarian cancer, and ^211^At and ^212^Pb conjugated to antibodies are in clinical development (19–22). Preclinical and clinical data indicate that α-emitters are well tolerated without dose-limiting toxicity (23, 24).

Radspherin® is a novel treatment principle especially designed to give local radiation to the surface of the abdominal cavity based on biodegradable microparticles with ^224^Ra adsorbed to the particle. By injection into the peritoneal cavity, the particles are distributed and emit internal α-particle radiation to the tissue of the peritoneal lining and potentially kill remaining free cancer cells and small cell clusters and hopefully will prevent the further spread of disease.

In this study, we report our first experience from a phase 1 study in patients with PM-CRC to evaluate the safety and toxicity of Radspherin®, determine the recommended, and/or establish a recommended dose for Radspherin® as a single IP or two repeated doses following CRS-HIPEC.

## Materials and methods

### Approval

The study was approved by the National Ethics Committees in Norway and Sweden, the Norwegian Medicines Agency, and the Swedish Medical Products Agency. Data were registered in the Sponsors database (Viedoc eCRF).

### Patients and surgical treatment

A first-in-human, phase 1 study (EudraCT 2018–002803-33) was conducted at two specialized CRS-HIPEC centers in Oslo, Norway, and Uppsala, Sweden. Twenty-three patients were included between 11 May 2020 and 16 August 2021. Twenty-nine patients were screened. CRS was performed to remove all macroscopically visible tumors, involving peritonectomy procedures and organ resections as necessary. Peritoneal tumor distribution was classified using the peritoneal cancer index (PCI) (25), and the completeness of cytoreduction (CC) score (25) was used to evaluate residual tumor after CRS. All CC-0 cases were given HIPEC. All anastomoses were completed before the HIPEC procedure.

The synchronous PM was defined as a diagnosis at or within 6 months of primary surgery, and disease-free interval (DFI) was the time from primary surgery to diagnosis of PM. Postoperative complications (30-day morbidity and mortality) were classified according to Accordion (26).

### Hyperthermic intraperitoneal chemotherapy

Hyperthermic intraperitoneal chemotherapy was administrated using a closed technique with an open abdomen in Norway (27), whereas the closed abdomen technique was used in Sweden (28). In Norway, the HIPEC regimen contained mitomycin, 35 mg/m^2^ (maximum 70 mg), given in three fractions for 90 min (50% initially, 25%/30 min, and 25%/60 min), whereas in Sweden, oxaliplatin 460 mg/m^2^ or irinotecan 460 mg/m^2^ were both given in 30 min.

#### Catheter insertion

Following the CRS-HIPEC, an in-dwelling peritoneal Blake catheter was placed anteriorly in the upper abdominal cavity. The catheter was obliquely tunneled, clamped, and fixed to the abdominal wall to reduce the risk of leakage or displacement.

#### Study design and administration of Radspherin®

**Figure 1:**
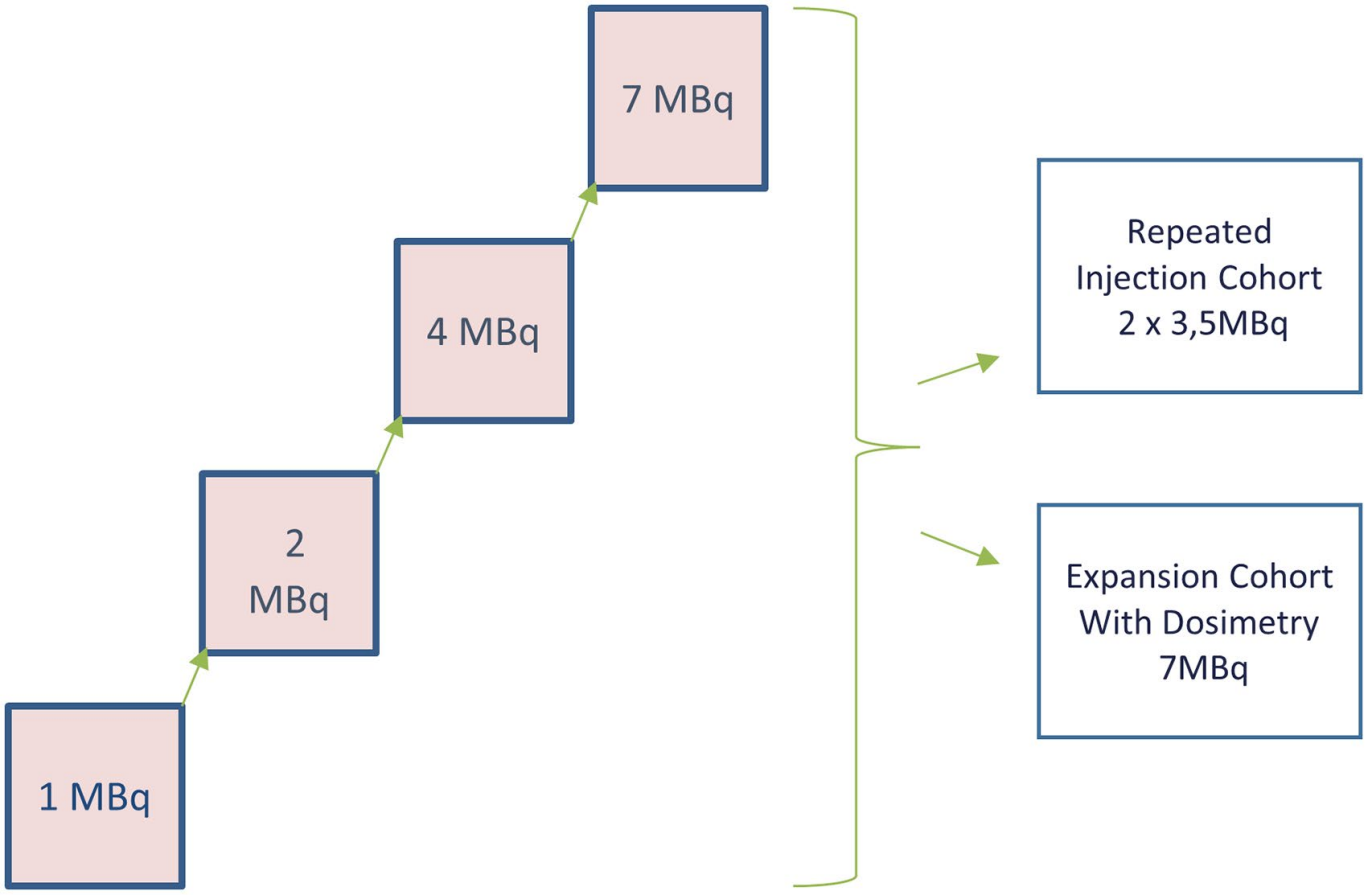
Dose escalation aimed to define the recommended dose on 3 + 3 subjects (12). Thereafter, a repeated injection cohort (3) and an expansion cohort were performed (6).

The dose escalation was performed as a 3 + 3 design (Figure 1), increasing dose levels starting at 1 MBq followed by 2, 4, and 7 MBq or until eventual dose-limiting toxicity (DLT) was observed. The repeated injection cohort included three subjects for the highest dose level that has been declared safe (explored as a split dose of two separate injections given 1 week apart). The study also involved an expansion cohort with six subjects at the highest safe activity dose safe. Radspherin® was injected in the abdominal cavity through a catheter 2 days after CRS-HIPEC for patients to have stabilized after the complex surgery. Each subject was followed until disease progression in the abdominal cavity or for 12 months (18 months after the highest dose level) after the administration of Radspherin®. The results from the safety interim analysis after the completion of the pre-defined DLT period of 30 days are presented.

Dose-calibrated Radspherin® (up to 10 mL containing 0.7–1 g of particles) was prepared at the nuclear medicine department at the site and administered as a single bolus injection *via* a three-way Luer lock connected to the inserted peritoneal catheter. After the injection, the catheter was flushed with about 250 ml of isotonic solution, and in all instances, all drains were kept clamped for a minimum of 72 h, except in one patient where a laparotomy was performed after 65 h. The patient moved from side to side in the bed regularly for the first 2 h after installation. For repeated injections, the same in-dwelling peritoneal catheter was used and then removed 3–4 days later.

The peritoneal distribution of Radspherin® particles was examined by single-photon emission computed tomography/computed tomography (SPECT/CT) gamma-camera imaging performed on days 1, 2, and 3 (Day 6 for the dosimetry cohort). The patients were followed closely during the hospital stay and later at pre-scheduled intervals to discover complications such as suspected unexpected serious adverse reactions (SUSARs), serious adverse effects (SAEs), and adverse effects (AEs). The EMA “Guideline on strategies to identify and mitigate risks for first-in-human and early clinical trials with investigational medicinal products” (EMEA/CHMP/SWP/28367/07 Rev. 1) has been considered for the assessment of factors of risk.

### Study objectives

The primary objectives of the study were to investigate the safety and toxicity of Radspherin® and to determine the recommended dose of Radspherin®, among the four suggested doses of 1, 2, 4, and 7 MBq following CRS and HIPEC (Figure 1).

The secondary objectives of the study were to establish a recommended dose of Radspherin® as a single IP injection or two repeated IP injections following CRS and HIPEC and to describe the biodistribution of Radspherin®.

### Additional systemic chemotherapy

According to national guidelines, adjuvant chemotherapy was not routinely given. In the case of synchronous PM with locoregional lymph node metastasis, adjuvant chemotherapy was recommended after CRS-HIPEC/Radspherin®, otherwise not.

### Data analysis

All data were recorded in the eCRF, and external study monitoring and source data verification were performed. The study was reviewed by an Independent Data Monitoring Committee. Categorical variables were described using frequencies/percentages, and continuous variables were described with median/range. Safety evaluations were based on the incidence, intensity, and type of AEs, and clinically significant changes in the subjects’ vital signs and clinical laboratory results.

## Results

**Figure 2:**
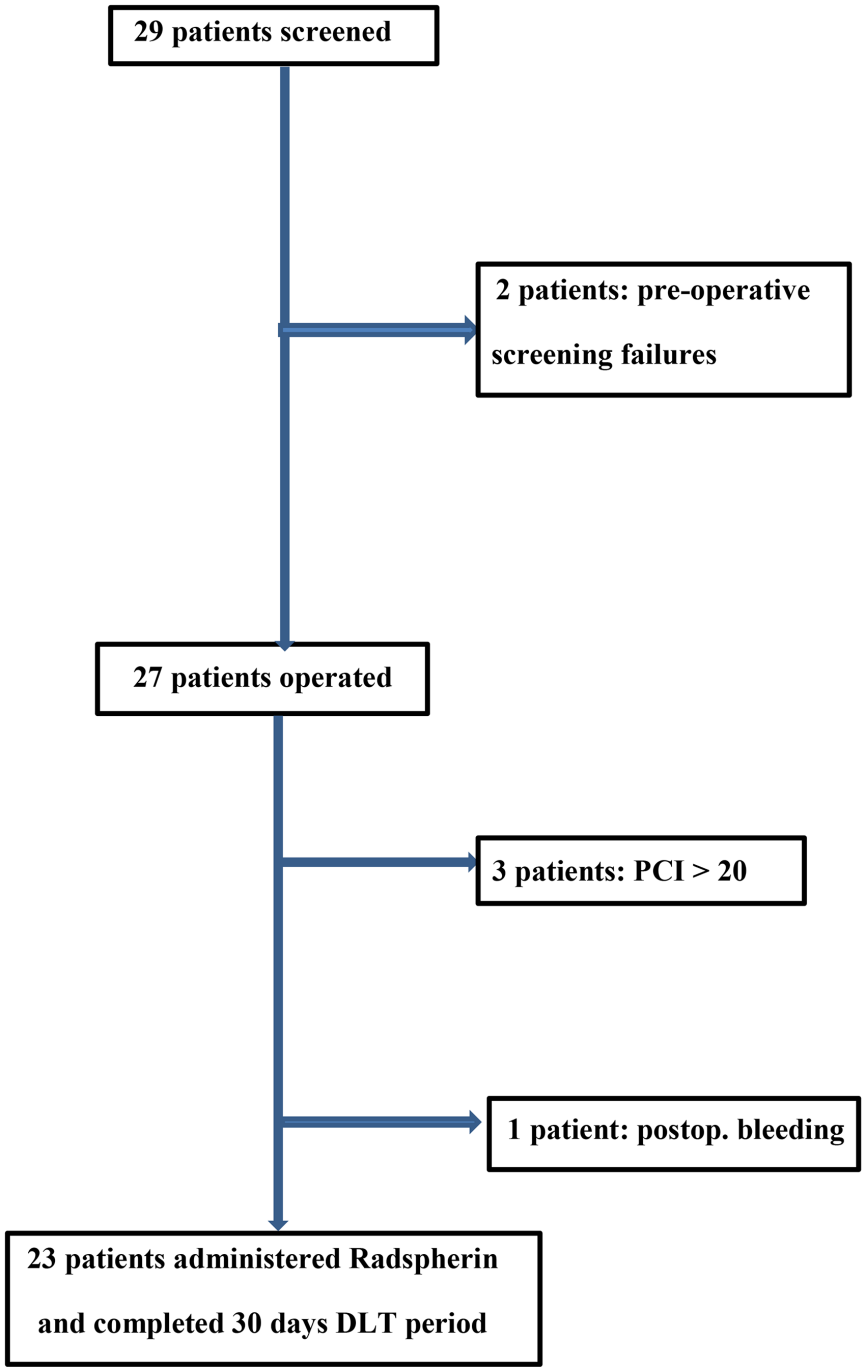
Consort flow diagram of the study patients (*n* = 23).

Twenty-nine patients were screened for the study (Figure 2). Totally, there were six screening failures due to the extent of metastasis (PCI > 20; 3), other previous malignant diseases (2), or peroperative bleeding (1) leading to exclusion from the study before the decision on giving Radspherin®. Accordingly, 23 patients were given Radspherin®. Of the 23 patients, 19 patients were treated at Oslo University Hospital and four at Uppsala Academic Hospital in Sweden. The study had a dose escalation cohort (14 pts.) with increasing doses from 1 MBq (4 pts.) to 2 MBq (3 pts.), 4 MBq (4 pts.), and 7 MBq (3 pts.), a repeated cohort (3 pts.) with 3.5 MBq given two times with 1-week interval, and an expansion cohort on highest dose level 7 MBq with additional six patients (Figure 1).

Table 1 summarizes the clinicopathological characteristics of the study cohort, which comprised 16 women (70%) and seven men (30%) with a median age of 64 years (28–78). Twelve patients were diagnosed with IUCC stage IV disease after primary surgery. Metachronous metastasis occurred after a disease-free interval (DFI) of median of 11 months (range 3–30). Approximately 43% had received chemotherapy at some point before CRS-HIPEC. Performance status was in most cases ECOG 0, while only one patient was in ECOG 1. One patient in the 7 MBq cohort received neoadjuvant irradiation therapy. Lymph node metastasis was present in 15 patients (65%) of the primary cases.

**Table 1:** Clinicopathological characteristics after CRS-HIPEC (*n* = 23).

	Dose escalation and expansion cohorts				Repeat injection cohort	
	1 MBq	2 MBq	4 MBq	7 MBq	2 × 3.5 MBq	Total
	*N* = 4	*N* = 3	*N* = 4	*N* = 9	*N* = 3	*N* = 23
Age, years						
Median	58.0	72.0	68.0	61.0	71.0	64.0
Min, Max	44, 71	69, 74	56, 78	28, 68	42, 78	28, 78
Sex, *n* (%)						
Male	2 (50%)	0	1 (25%)	2 (22%)	2 (67%)	7 (30%)
Female	2 (50%)	3 (100%)	3 (75%)	7 (78%)	1 (33%)	16 (70%)
Stage, *n* (%)						
Stage II	0	2 (67%)	1 (25%)	1 (11%)	2 (67%)	6 (26%)
Stage III	1 (25%)	1 (33%)	0	3 (33%)	0	5 (22%)
Stage IV	3 (75%)	0	3 (75%)	5 (56%)	1 (33%)	12 (52%)
Metachr mets	1	3	1	4	2	11
DFI						
Median (mnt)	10	19	13	11	11	15
Min, Max	10	11,25	13	3,17	6,16	3,30
ECOG performance status						
Grade 0	4 (100%)	3 (100%)	4 (100%)	8 (89%)	3 (100%)	22 (96%)
Grade 1	0	0	0	1 (11%)	0	1 (4%)
Prior chemotherapy, n (%)						
Yes	2 (50%)	1 (33%)	1 (25%)	4 (44%)	2 (67%)	10 (43%)
No	2 (50%)	2 (67%)	3 (75%)	5 (56%)	1 (33%)	13 (57%)
						
LN +	3 (75%)	1 (33%)	3 (100%)	7 (78%)	1 (33%)	15(65%)
Median	2	0	3	1	**	4
Min,Max	2,11	0,14	0,13	0,19	**	1, 19

*N*, number of patients in the analysis set; *n*, number of patients meeting the criterion; Max, maximum; Min, minimum; DFI, disease-free interval from surgery for a metachronous primary tumor; LN+, number of lymph node metastases; ^**^N1c.

The median PCI at the time of CRS-HIPEC was 7 (3–19; Table 2). The median duration of surgery was 374 min (266–508). The median peroperative bleeding was 300 mL (50–1,000 mL). In-hospital time was 11 days (7–37). At Norwegian Radium Hospital, HIPEC is performed with mitomycin C, 35 mg/m^2^ up to 70 mg, median 63 mg (57–70), and given in a closed perfusion circuit with open abdomen; duration 90 min; and intra-abdominal temperature median 42.0°C. In Uppsala, oxaliplatin 460 mg/m^2^ or irinotecan 460 mg/m^2^ was perfused for 30 min. Accordingly, the total operation time was reduced by 60 min compared to the Norwegian site. The knife time is then median more than 4 h before HIPEC in this study with complex surgery for PM.

**Table 2:** Characteristics after CRS-HIPEC (*n* = 23).

	Dose escalation and expansion cohorts				Repeat injection cohort	
	1 MBq	2 MBq	4 MBq	7 MBq	2 × 3.5 MBq	Total
	*N* = 4	*N* = 3	*N* = 4	*N* = 9	*N* = 3	*N* = 23
PCI						
Median	10.5	7	11.5	8	6	7
Min Max	6,19	6,14	4,19	3,17	5,16	3,19
Blood loss (mL)						
Median	550	500	250	300	200	300
Min,Max	200,1,000	100,500	50,500	50,500	75,300	50,1,000
Duration of surgery						
Median	426	380	410	410	280	374
Min Max	374–485	372–480	330–500	301–508	266–288	266–508
HIPEC with Mitomycin C					-	
Median (mg)	70	61	64	62	-	63
Min,Max	70.70	60,63	59,70	57,70	-	57,70
HIPEC in Sweden^*^						
Median (mg)	-	-	Comment^*^	-	Comment^*^	-
Min,Max	-	-	-	-	-	-
Hospital stay						
Median	9.5	7	16	11	16	12
Min,Max	8,16	7,21	9,37	8,16	15,16	7,37
Accordion						
Median	1.5	1	2.5	1	2	2
Min,Max	1,4	1,3	1,4	1,2	2,2	1,4

*N*, number of patients in the analysis set; Max, maximum; Min, minimum; PCI, peritoneal cancer index; and duration of surgery, knife time.

* HIPEC in Sweden: Patient 4 MBq oxaliplatin 620 mg/30 min. Patients repeat injection cohort; irinotecan 960 mg/90 min or oxaliplatin 920 or 760 mg/30 min.

The highest dose escalation level 4, the 7 MBq dose, was selected as recommended dose, as no DLT was observed. The incidence of DLTs, TEAEs, and SAEs is summarized in Table 3. The actual amount of Radspherin® administered is shown in Table 4. All 23 patients were included in the safety population. A total of 68 TEAEs were reported for 17 patients (74%) during the first 30 days. Of these, 23 of grade 2 before Radspherin® installation and 45 of grade 2 in the time period after Radspherin® installation (Days 1–30) were reported in 16 patients. There was one grade 3 TEAE which was reported as SAE but unrelated to Radspherin®. The most frequently reported AEs were vomiting, pyrexia, nausea, and decreased appetite, and the majority were considered related to CRS and/or HIPEC. Only six of the TEAEs were evaluated as related to Radspherin® and laboratory test abnormalities [platelet count increased, blood alkaline phosphatase increased, hemoglobin decreased (*n* = 2), monocyte count increased, and hepatic enzyme abnormal]. All these TEAEs were resolved with no actions taken and no need for additional treatment.

**Table 3:** Number of treatment-emergent adverse events in the time period of 1–30 days.

	Dose escalation and expansion cohorts				Repeat injection cohort	
	1 MBq	2 MBq	4 MBq	7 MBq	2 × 2.5 MBq	Total
	*N* = 4	*N* = 3	*N* = 4	*N* = 9	*N* = 3	*N* = 23
	E, n (%)	E, n (%)	E, n (%)	E, n (%)	E, n (%)	E, n (%)
TEAE of CTCAE Grade 2	4, 2 (50%)	3, 1 (33%)	17, 4 (100%)	18, 8 (89%)	3, 1 (33%)	45, 16 (70%)
TEAE of CTCAE Grade ≥ 3	0, 0	0, 0	1, 1 (25%)	0, 0	0, 0	1, 1 (4%)
SAE	1, 1 (25%)	1, 1 (33%)	2, 1 (25%)	0, 0	0, 0	4, 3 (13%)
DLT	0, 0	0, 0	0, 0	0, 0	0, 0	0, 0

E, number of adverse events after the administration of Radspherin; *N*, number of patients in the analysis set; *n*, number of patients meeting the criterion; TEAE, treatment-emergent adverse event, SAE, serious adverse event; DLT, dose-limiting toxicity.

**Table 4:** Administered dose and compliance.

	Dose escalation and expansion cohorts				Repeat injection cohort
	1 MBq	2 MBq	4 MBq	7 MBq	2 × 2.5 MBq
	*N* = 4	*N* = 3	*N* = 4	*N* = 9	*N* = 3
Administered Dose, Radspherin® MBq					
Median	1.01	2.05	3.92	7.15	7.06
Min, Max	0.98, 1.07	1.98, 2.09	3.74, 4.05	6.99, 7.36	7.05, 7.20

*N*, number of patients in the analysis set; Max, maximum; and Min, minimum.

Four SAEs within 30 days were reported for three patients, and all were considered unrelated to Radspherin®. These SAEs included one anastomotic leak (grade 3), which was reoperated on Day 2, two abdominal infections (grade 2) that required a drain on Day 10, and an anastomotic leak that required reoperation on Day 10 and a drain on Day 15 (see the section “Discussion”). During reoperations, abdominal fluid was drained before opening the abdomen, washed out with physiological saline solution liquid, and removed as irradiation waste. No patients in the repeat injection cohort had any SAE. No deaths or study discontinuations due to TEAEs or SAEs were reported during the 30 days.

**Figure 3:**
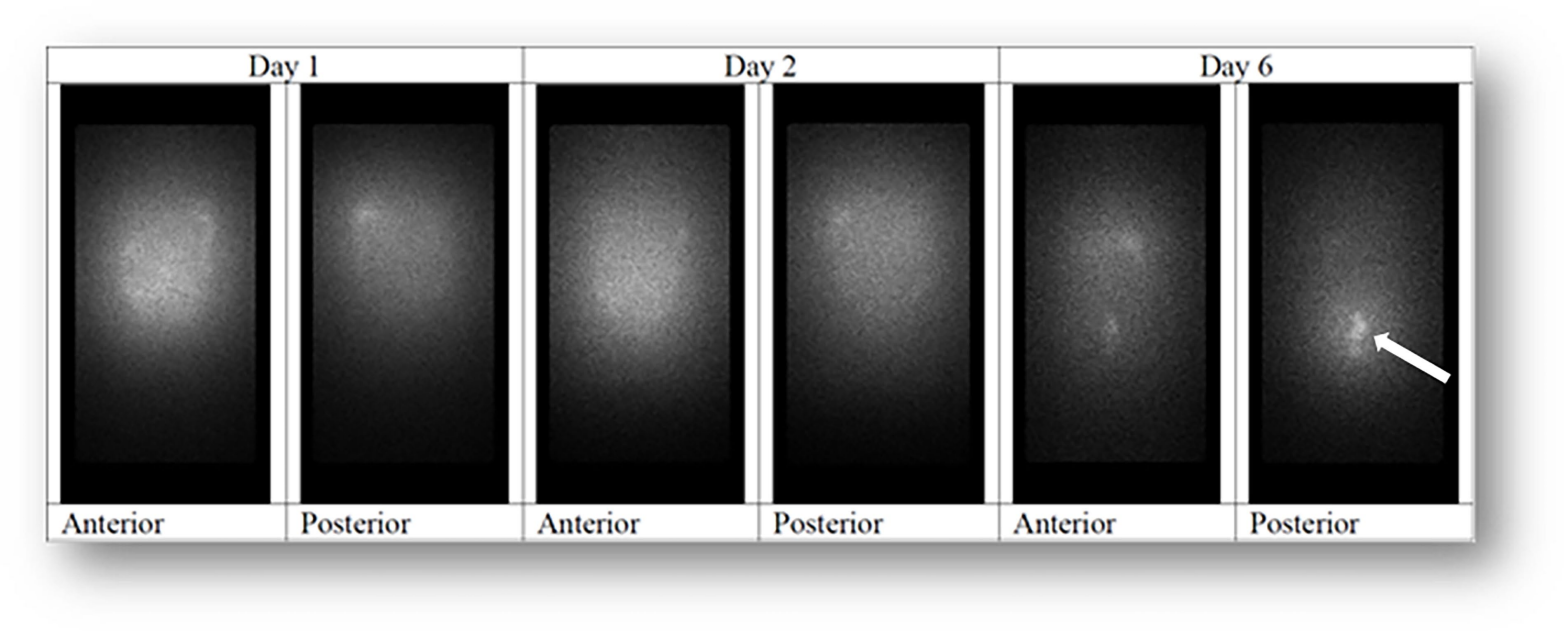
^224^Ra-labeled microparticles for patient at 7 MBq were evenly distributed in the abdominal cavity both in the anterior and posterior images. In this subject, an area with a slightly higher activity was observed in the left upper region. No areas with low levels of activity were observed. At a late time point, uptake was observed in the distal large intestine (arrow).

Corresponding Accordion grade 3 events occurred in two of the 23 patients (draining of abscesses) and Accordion grade 4 events in two (reoperation due to anastomotic leaks; Table 3). There were no deaths within 100 days. The biodistribution of Radspherin® showed a relatively even peritoneal distribution, and an example is shown in Figure 3.

## Discussion

The CRS-HIPEC procedure is well known to be associated with postoperative complications (29), and significantly higher incidences of severe postoperative complications (i.e., fistulas and anastomotic leaks) have been observed in patients treated with HIPEC than in patients treated without HIPEC (30).

In the current study, there was no 30-day mortality. The incidence of severe postoperative complications (Accordion 3), the need for drainage or parenteral nutrition occurring in five of the 23 patients (22%), and the reoperation rate of 9% (two of 23 patients) were all as expected and suggest that the treatment with Radspherin® is well tolerated and safe. The first patient with anastomotic leakage in the study experienced an increase in white blood cells to 18.7 × 10^9^/L and a moderate elevation of C-reactive protein (CRP) to 61 the day after surgery and the day before Radspherin® installation, followed by antibiotics the next day and reoperation with the verification of anastomotic leakage 2 days after Radspherin®. The other patient also experienced an increase in white blood cells to 16.7 × 10^9^/L and a moderate CRP increase to 58 the day after surgery and received Radspherin® the following day. Five days later, intravenous antibiotics were started due to an infection. Anastomotic leakage was diagnosed on Day 10, and a laparotomy with resection and stoma was performed. Both cases were considered caused by infection before Radspherin® and to be related to the CRS and HIPEC procedures.

In other larger patient series, postoperative mortality between 0.7 and 7.7% has been reported (29, 31, 32) with reoperation rates varying between 4 and 20.8% (13). Oslo University Hospital has previously reported corresponding numbers of 0% (mortality), 15% (Accordion ≥3), and 8% (reoperation rate) (14) with CRS-HIPEC and without Radspherin®.

Norwegian Radium Hospital recently performed a dose-escalating phase I trial with intraperitoneal (IP) MOC31PE immunotoxin in PM-CRC after CRS-HIPEC (33) showing promising results for better control of PM. The hospital has used radium and α-emitters for the treatment of metastatic cancer with Xofigo®, a ^223^Ra radiopharmaceutical. Xofigo® was approved by the FDA and EMEA in 2013 for the treatment of symptomatic bone metastasis from prostate cancer.

Because of the short range and high linear energy transfer of α-particle emitters, there is a much higher relative biological effectiveness of the radiation from Radspherin® than from β-particle emitting radiopharmaceuticals previously used. Thereby, α-particle emitters are theoretically more efficient in treating micrometastases and killing chemotherapy-resistant tumor cells. The much shorter radiation range prevents the radiation of tissue in deeper regions of sensitive abdominal organs (i.e., small intestine), which was the prime reason for abandoning the β-particle emitting radiopharmaceuticals, giving a discrete surface irradiation of just the serosal lining of the peritoneal cavity.

This favorable safety profile in the current study is in line with documentation from other preclinical and clinical studies with other related alpha-emitting compounds administered intraperitoneally. Safety and effect of IP administration have been demonstrated in animal models both with colloids/particles and antibodies as carriers of a range of radionuclides: ^211^At polymers (34, 35), bismuth-213 (^213^Bi) antibodies (36), ^211^At antibodies (37, 38), ^212^Pb antibodies (21, 39, 40), thorium-227 (^227^)Th antibodies (41), and actinium-225 (^225^Ac) antibodies (42).

All dose levels of Radspherin® were well tolerated with DLT not reached. No deaths occurred, and no SAEs were considered related to Radspherin®. The biodistribution of Radspherin® showed a good peritoneal distribution of the radiolabeled microparticles. Long-term safety, dosimetry, and first efficacy results of Radspherin® will be reported after 18 months of the follow-up period.

## Conclusion

All dose levels of Radspherin® were well tolerated with DLT not reached. No deaths occurred, and no SAEs were considered related to Radspherin®. The biodistribution of Radspherin® showed a good peritoneal distribution of the radiolabeled microparticles.

## Data availability statement

The raw data supporting the conclusions of this article will be made available by the authors, without undue reservation.

## Ethics statement

The studies involving human participants were reviewed and approved by National Ethics Committees in Norway and Sweden. The patients/participants provided their written informed consent to participate in this study.

## Author contributions

SL, M-ER, and ØB: conceptualization. SL, WG, M-ER, and ØB: study design and drafting the manuscript and revising it critically for important intellectual content. SL, WG, and ØB: data analysis. SL, WG, SS, NL, AH, M-ER, and ØB: interpretation of results. All authors contributed to the article and approved the submitted version.

## Funding

This study was funded by Oncoinvent AS and Innovation Norway.

## Conflict of interest

ØB is a clinical consultant to and holds ownership in Oncoinvent AS.

The remaining authors declare that the research was conducted in the absence of any commercial or financial relationships that could be construed as a potential conflict of interest.

The authors declare that this study received funding from Oncoinvent AS. Oncoinvent AS was involved in the study design, collection, analysis, interpretation of data and the decision to submit this article for publication.

## Publisher’s note

All claims expressed in this article are solely those of the authors and do not necessarily represent those of their affiliated organizations, or those of the publisher, the editors and the reviewers. Any product that may be evaluated in this article, or claim that may be made by its manufacturer, is not guaranteed or endorsed by the publisher.
